# Comparison of Size and Geography of Airborne Tungsten Particles in Fallon, Nevada, and Sweet Home, Oregon, with Implications for Public Health

**DOI:** 10.1155/2012/509458

**Published:** 2012-03-14

**Authors:** Paul R. Sheppard, Brian J. Bierman, Kent Rhodes, Gary Ridenour, Mark L. Witten

**Affiliations:** ^1^Laboratory of Tree-Ring Research, University of Arizona, Tucson, Az 85721, USA; ^2^McCrone Associates, Inc., 850 Pasquinelli Drive, Westmont, IL 60559, USA; ^3^625 W. Williams, Suite B, Fallon, Nevada 89406, USA; ^4^Odyssey Research Institute, 7032 East Rosewood Street, Tucson, AZ 85710, USA

## Abstract

To improve understanding of possible connections between airborne tungsten and public health, size and geography of airborne tungsten particles collected in Fallon, Nevada, and Sweet Home, Oregon, were compared. Both towns have industrial tungsten facilities, but only Fallon has experienced a cluster of childhood leukemia. Fallon and Sweet Home are similar to one another by their particles of airborne tungsten being generally small in size. Meteorologically, much, if not most, of residential Fallon is downwind of its hard metal facility for at least some fraction of time at the annual scale, whereas little of residential Sweet Home is downwind of its tungsten facility. Geographically, most Fallon residents potentially spend time daily within an environment containing elevated levels of airborne tungsten. In contrast, few Sweet Home residents potentially spend time daily within an airborne environment with elevated levels of airborne tungsten. Although it cannot be concluded from environmental data alone that elevated airborne tungsten causes childhood leukemia, the lack of excessive cancer in Sweet Home cannot logically be used to dismiss the possibility of airborne tungsten as a factor in the cluster of childhood leukemia in Fallon. Detailed modeling of all variables affecting airborne loadings of heavy metals would be needed to legitimately compare human exposures to airborne tungsten in Fallon and Sweet Home.

## 1. Introduction

**Figure 1:**
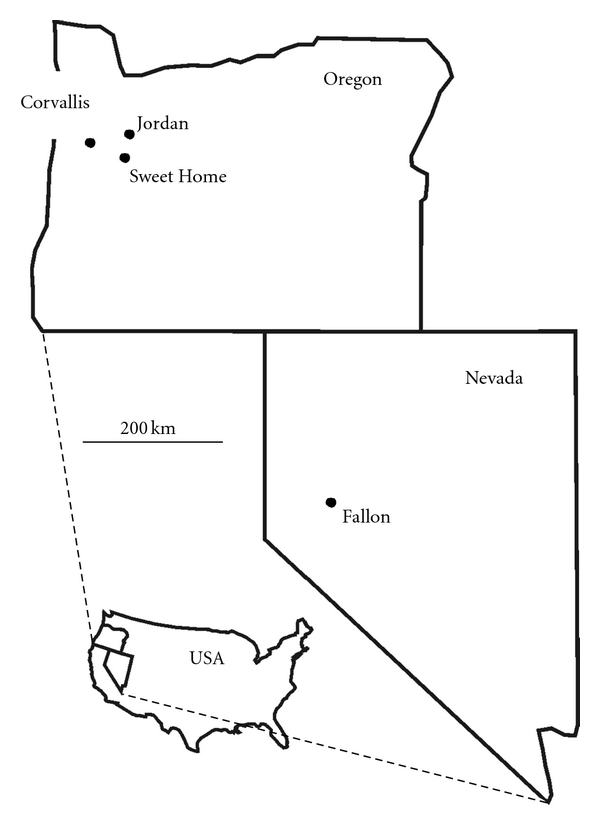
Regional map of Nevada and Oregon showing the location of Fallon and Sweet Home and weather stations of Oregon.

Size and geography of airborne tungsten particles collected in Fallon, Nevada, and Sweet Home, Oregon (Figure 1), were compared as part of ongoing research on the cooccurrence of airborne tungsten and a cluster of childhood leukemia in Fallon. Fallon experienced a cluster of childhood leukemia beginning in 1997 [3], with the last case announced in 2004 [4]. Although the cluster is thought to have abated [5], at least one additional case of childhood leukemia has occurred in Fallon since 2004 [6]. Given Fallon's pediatric population of about 2500 children up to 19 years in age [1], and a national expected rate of childhood leukemia of 4.1 cases per 100,000 children up to 19 years in age per year [7], the expected rate of childhood leukemia for Fallon should be only one case every ten years.

This cluster, deemed “one of the most unique … ever reported” [8], prompted extensive research in an effort to find a cause. Among other findings, multiple lines of evidence have shown that Fallon has elevated levels of airborne tungsten and cobalt [9–13].

Although Nevada is naturally rich in tungsten minerals, including geologically [14] and hydrologically [15, 16], Fallon also has a potential anthropogenic source of airborne tungsten. An industrial facility specializing in hard-metal metallurgy, which uses tungsten carbide and cobalt to produce tool materials [17], is located within Fallon. This hard-metal facility was named by the Nevada State Health Division as a candidate source of tungsten in Fallon [18]. Morphological and chemical characteristics of airborne tungsten particles in Fallon indicate that they are anthropogenic in origin, not natural [19].

 To improve understanding of possible connections between airborne tungsten and public health in Fallon, it would be useful to replicate this research in other small communities that have an industrial source of airborne tungsten in order to compare airborne tungsten and rates of cancer across towns. Sweet Home, Oregon, is another town that has an industrial source of airborne tungsten. As a first step in comparing Sweet Home to Fallon, environmental monitoring techniques used in Fallon were employed in Sweet Home [20]. Elevated airborne tungsten was accurately indentified near the known industrial facility in Sweet Home relative to outlying forests and to the outskirts of Sweet Home.

To our knowledge, Sweet Home has not experienced increased rates of cancer. This prompts a question about airborne tungsten and public health: if exposure to airborne tungsten and/or cobalt particles caused, or even contributed to, the cluster of childhood leukemia in Fallon, then why is there not an increased rate of childhood leukemia in Sweet Home? One possible answer to this question could be that tungsten particles and/or geographical traits differ between Fallon and Sweet Home such that actual human exposure to airborne tungsten differs between these towns. Accordingly, the objectives of this research were (a) to measure and characterize the size of airborne tungsten particles of both towns, (b) to analyze spatial patterns of dispersal of airborne tungsten of both towns, and (c) to compare potential human exposure to airborne tungsten between Fallon and Sweet Home.

## 2. Materials and Methods

### 2.1. Fallon and Sweet Home

Fallon and Sweet Home are similar in that both are rural towns with small populations of about 8,000 people (Table 1), and both towns have industrial facilities that process or otherwise use fine tungsten particles. Based on number of employees, the Fallon tungsten facility is larger than that of Sweet Home. Fallon and Sweet Home have similar annual mean temperatures (~11°C), but Sweet Home receives 10 times more rainfall than Fallon on average.

### 2.2. Air Sampling

In March and November, 2004, airborne dust was collected within Fallon using portable, high-volume particulate air samplers [9]. Weather during these collection periods was generally sunny and windy in March and rainy in November. The filter type was glass-fiber, a common medium for high-volume sampling of airborne particulates [21, 22]. Filters were 510 *μ*m thick and had up to 99.99% retention for particles down to sub-*μ*m sizes [23]. In May, 2005, airborne dust was collected within Sweet Home using the same equipment used in Fallon [20]. Weather during this collection period was generally sunny and calm.

For the particle size part of this research, three filters were selected from both Fallon and Sweet Home for further measurement and analysis. The filters were selected to optimize a transect of distance from their respective industrial tungsten facilities.

### 2.3. Additional Sampling in Sweet Home

Two additional samples of tungsten-laden dust were collected in 2011 in Sweet Home. One, dust was collected from the powder drum itself, which was not the actual product of the industrial facility but rather the fine waste that results from its processing. This allowed assessment of tungsten particles that result from the production process. Two, surface dust was swept up from pavement just east of the facility. This allowed assessment of airborne tungsten particles that drift out of the building but do not travel far from the source.

### 2.4. Isolation of Tungsten Particles

To remove the collected particulate matter from the glass-fiber filters for microanalysis, a 20 cm^2^ portion of each filter was placed into its own 50 mL plastic centrifuge tube with approximately 10 mL of ethanol. The tubes were sonicated for 20 minutes to dislodge the particles, and then the filter pieces were removed from the tubes and saved. Approximately 50 mg of the powder drum and surface dust samples was placed into their own centrifuge tubes, again with approximately 10 mL of ethanol.

Fifteen mL of methyl iodide was added to the centrifuge tubes, and the samples were centrifuged for 10 minutes at 2000 rpm. The ethanol layer was pipetted off and saved. The bottom methyl iodide layer was filtered on 25-millimeter polyester membrane filters and mounted onto aluminum stubs for automated analysis. This method recovers a representative sample for particle sizing and chemical analysis and has worked specifically with tungsten particles in prior work [19].

### 2.5. Automated Particle Analysis

Samples were analyzed using an ASPEX 3025 personal scanning electron microscope (PSEM) utilizing energy dispersive X-ray spectrometry (EDS) and ASPEX's automated feature analysis (AFA) software. This system and software located, counted, measured, and quantitatively analyzed particles in fully automated mode. Particles containing less than 80% tungsten were culled out of the data set. Frequency histograms plotting the size of tungsten particles were generated, and images of representative particles were collected. Calibration was performed using (1) a certified tungsten standard from Geller Microanalysis Laboratory for tungsten quantification, (2) a copper standard for energy scaling, and (3) a commercial standard (PGS) from Aspex LLC for particle sizing.

### 2.6. Geographical Analysis

Aerial photos of both towns were labeled with limits of residential areas, locations of respective industrial tungsten facilities, and circles of elevated levels of airborne tungsten. Prevailing wind directions of both towns were illustrated with wind rose diagrams using data from nearby weather stations.

## 3. Results and Discussion

### 3.1. Tungsten Particles from Air Filters

**Figure 2:**
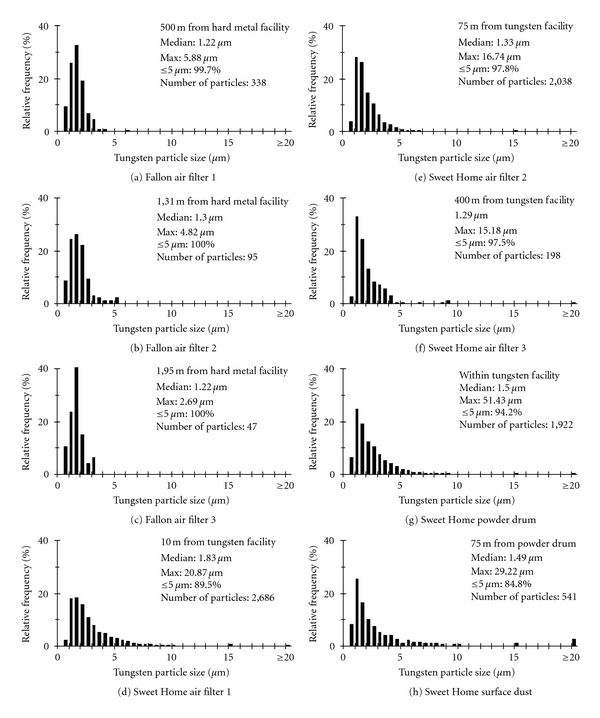
Frequency histograms of tungsten particles by size for each sample analyzed from Fallon and Sweet Home.

The size distributions of tungsten particles are similar across all six air filters from both towns. Median sizes of tungsten particles across all air filters range from 1.22 to 1.83 *μ*m in diameter (Figures 2(a)–2(f)). This size class (<2.1 *μ*m) is typical for airborne particulates of heavy metals [24]. The particulate size class of 1 to 2 *μ*m in diameter is also considered seriously threatening to human health [25]. Additionally, the vast majority of tungsten particles isolated from both towns air filters were below 5 *μ*m in size (Figures 2(a)–2(f)). The Sweet Home filters also contained tungsten particles considerably larger than the median size, ranging up to 21 *μ*m in diameter, but very particles were this large.

### 3.2. Tungsten Particles from the Powder Drum and Surface Dust in Sweet Home

The vast majority of tungsten particles collected from the powder drum and surface dust samples of Sweet Home were below 5 *μ*m in size (Figures 2(g)-2(h)). Median particle sizes were ~1.50 *μ*m in both cases. In general, the size distributions of tungsten particles of these nonairborne samples are similar to the airborne samples of Sweet Home, illustrating that airborne tungsten particles collected with air filters accurately reflect the size distribution of tungsten particles at the industrial source.

Maximum particle size from the drum sample was just over 50 *μ*m (Figure 2(g)), accurately reflecting the large target particle size of the manufacturer (personal communication with the facility manager). Maximum particle size from the surface dust sample was smaller (Figure 2(h)), just under 30 *μ*m, accurately reflecting that large, dense airborne particles do not travel as far as smaller particles [26].

### 3.3. Geographical Location of Tungsten Facilities Relative to Their Towns

**Figure 3:**
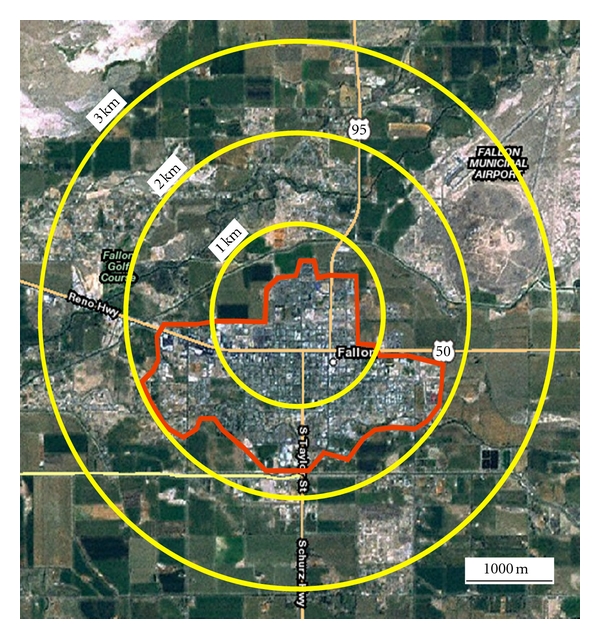
Aerial view of Fallon, Nevada, with 1 km distances centered on the hard metal facility. The red boundary marks the limit of residential Fallon.

Fallon is relatively circular in layout, more or less centered on the crossroads of two highways (Figure 3). The hard metal facility of Fallon is located just northwest of the crossroads. Airborne tungsten loadings within 3 km of the hard metal facility can be elevated above loadings farther away that can be considered as background levels [9]. Most of residential Fallon is within 3 km of the hard metal facility, and much of Fallon is within 2 km of it. Thus, most Fallon residents potentially spend time daily within an environment of elevated levels of airborne tungsten.

Interestingly, urine samples of Fallon residents were significantly elevated in tungsten [27]. No linkage was concluded between elevated tungsten in Fallon residents and leukemia occurrence, in part because people from both the case and control populations showed elevated tungsten levels, dismissing tungsten as a discriminating factor for occurrence of leukemia. This inability to conclude linkage does not rule out linkage but rather reflects the difficulty of conclusively establishing linkage using the case-comparison study design [28].

**Figure 4:**
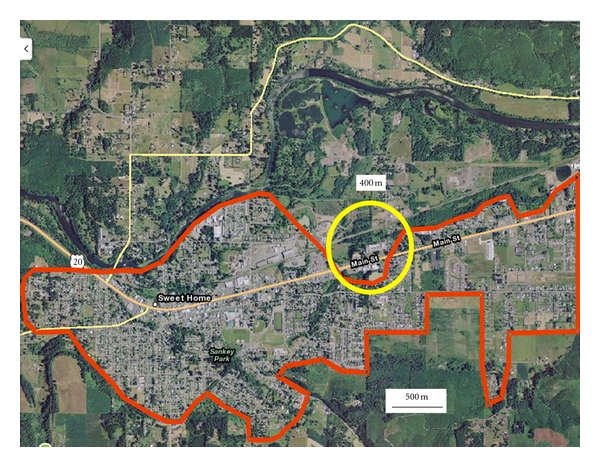
Aerial view of Sweet Home, Oregon, with the 400-m distance centered on the tungsten facility. The red boundary marks the limit of residential Sweet Home.

In contrast to the roughly circular layout of Fallon, Sweet Home is relatively linear, stretching out along a single highway (Figure 4). The tungsten facility of Sweet Home is located just east of the center of town, on the northern side. Airborne tungsten loadings were elevated above background levels out to only 400 m away from the tungsten facility [20], a considerably shorter dispersal distance than the 3 km of Fallon. This could be explainable meteorologically: airborne heavy metals have been shown to correlate inversely with precipitation [29], and Sweet Home receives 10 times more rainfall than Fallon (Table 1). Little of residential Sweet Home lies within 400 m of the tungsten facility. Thus, few Sweet Home residents potentially spend time daily within an airborne environment with elevated levels of airborne tungsten. We know of no testing for tungsten in urine samples of Sweet Home residents to confirm exposure levels there.

### 3.4. Wind Patterns

**Figure 5:**
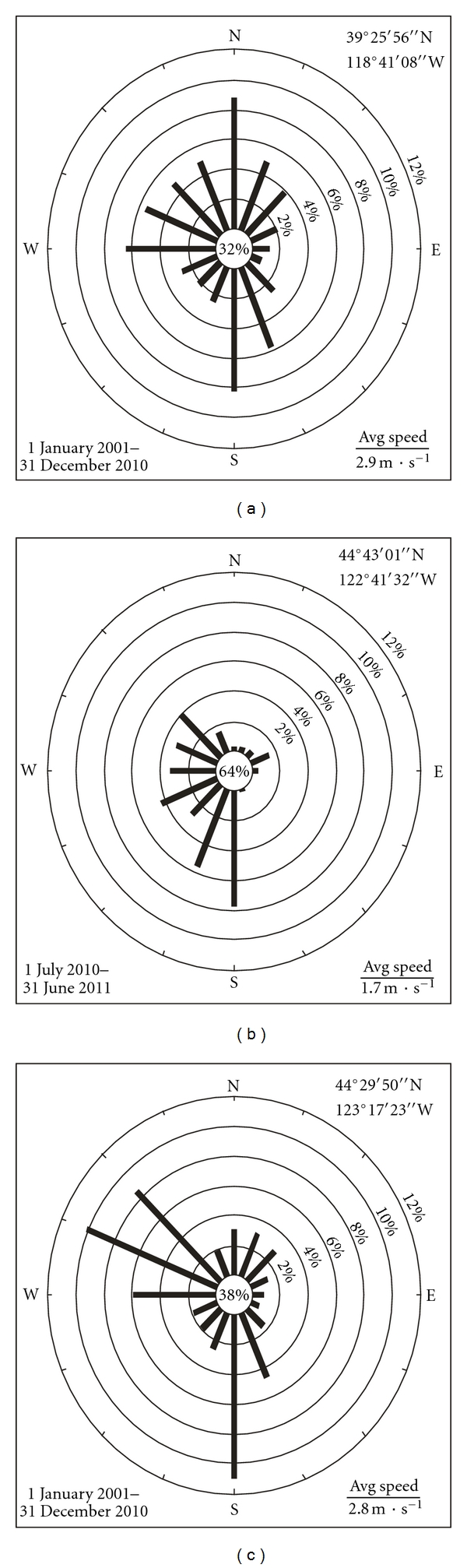
Wind rose diagrams for (a) Fallon, Nevada, a weather station with data for the entire decade of the 2000s, (b) Jordan, Oregon (Figure 1), a weather station with data for one year, and (c) Corvallis, Oregon (Figure 1), a weather station with data for the entire decade of the 2000s. Lines indicate directions that winds come from. Center percentages represent calm, that is, winds ≤ 2 m·sec^−1^. Time span and average wind speeds are given for each location. Wind data from NCDC, NOAA: wind roses generated using software of the Western Regional Climate Center.

Fallon typically experiences winds from the northeast, north, west, south, and southeast (Figure 5(a)). Given the central location of the hard metal facility in Fallon and these prevailing wind directions, much, if not most, of residential Fallon is downwind of the hard metal facility for at least some fraction of time at the annual scale. This should result in human exposure to elevated airborne tungsten levels for many, if not most, Fallon residents.

Sweet Home typically experiences winds from the northwest, west, southwest, and south (Figure 5(b)), or from the northwest and south (Figure 5(c)). Given the eastern location of the tungsten facility in Sweet Home and these prevailing wind directions, little of residential Sweet Home is downwind of the tungsten facility. This should result in little human exposure to elevated tungsten levels for Sweet Home residents.

## 4. Conclusions

As we have stated in prior work, it cannot be concluded from environmental data alone that elevated airborne tungsten causes childhood leukemia [9–13]. Such linkage requires direct biomedical research, which is at least supportable by the cooccurrence of exposure to airborne tungsten and a cluster of childhood leukemia [28, 30]. Tungsten has been evaluated for carcinogenicity, by itself [31, 32] as well as with other metals [33–36]. In general, this biomedical research has shown at least the possibility of linkage between exposure to tungsten and cancer.

Regardless of the toxicity of tungsten, this comparison of airborne tungsten and geography between Fallon and Sweet Home does lead to the following conclusion: the lack of excessive cancer in Sweet Home, which has an industrial tungsten facility as well as elevated levels of airborne tungsten, cannot logically be used to dismiss the possibility of airborne tungsten as a factor in the cluster of childhood leukemia in Fallon, which also has an industrial tungsten facility as well as elevated levels of airborne tungsten. The size distributions of airborne tungsten in each town are similar, but the relative sizes and locations of the tungsten facilities differ between Fallon and Sweet Home as do prevailing wind directions and annual precipitation amounts such that human exposure to airborne tungsten is probably higher in Fallon than in Sweet Home. Additional modeling of all variables affecting airborne loadings of heavy metals would be needed to legitimately compare human exposures to airborne tungsten in Fallon and Sweet Home. In any case, continued biomedical research on possible linkage of tungsten with leukemia is justified based on the cooccurrence of elevated airborne tungsten and a cluster of childhood leukemia in Fallon, Nevada.

## Figures and Tables

**Table 1:** Geographical comparison between Fallon and Sweet Home.

Community	Population^a^	No. of employees in tungsten facility	Annual temperature (°C)	Annual precipitation (mm)
Fallon	7,536	~100^b^	11.2	135
Sweet Home	8,016	11^c^	11.6	1,397

^a^From [1].

^b^From [2].

^c^Personal communication with facility manager.
